# Digital/computational phenotyping: What are the differences in the science and the ethics?

**DOI:** 10.1177/20539517211062885

**Published:** 2021-12-23

**Authors:** Federica Lucivero, Nina Hallowell

**Affiliations:** Ethox Centre and Wellcome Centre for Ethics & Humanities, Nuffield Department of Population Health, 6396 University of Oxford, UK

**Keywords:** Digital phenotyping, computational phenotyping, empistemology, affordances, digital biomarkers, ethics

## Abstract

The concept of ‘digital phenotyping’ was originally developed by researchers in the mental health field, but it has travelled to other disciplines and areas. This commentary draws upon our experiences of working in two scientific projects that are based at the University of Oxford’s Big Data Institute – The RADAR-AD project and The Minerva Initiative – which are developing algorithmic phenotyping technologies. We describe and analyse the concepts of digital biomarkers and computational phenotyping that underlie these projects, explain how they are linked to other research in digital phenotyping and compare and contrast some of their epistemological and ethical implications. In particular, we argue that the phenotyping paradigm in both projects is grounded on an assumption of ‘objectivity’ that is articulated in different ways depending on the role that is given to the computational/digital tools. Using the concept of ‘affordance’, we show how specific functionalities relate to potential uses and social implications of these technologies and argue that it is important to distinguish among them as the concept of digital phenotyping is increasingly being used with a variety of meanings.

This article is a part of special theme on Digital Phenotyping. To see a full list of all articles in this special theme, please click here: https://journals.sagepub.com/page/bds/collections/digitalphenotyping

## Introduction

Digital phenotyping is based on the assumptions that digital technologies can be used to identify phenotypic (observable physical or behavioural traits) features and this will facilitate better understanding of disease states. The concept of ‘digital phenotyping’ was originally developed by researchers in the mental health field (Onnela and Rauch, 2016), where the collection of data from wearable sensors and mobile phones has been seen as a way to acquire insight into people’s behaviours and habits in real time and in a more accurate way than using self-report questionnaires. Beyond its application in mental health research the idea of using digital technologies – including computing, machine learning techniques, smartphone applications, and wearable, environmental and mobile phone sensors – to collect and analyse environmental, behavioural and physiological data, explore phenotypic traits, and contribute to disease diagnosis, has driven much research and public health agendas (Huckvale et al., 2019; Waring and Majumder, 2020).

This commentary draws upon our experiences of working in two scientific projects that are based at the University of Oxford’s Big Data Institute – *https://www.radar-ad.org/* (Muurling et al., 2021) and *The Minerva Initiative* (Nellaker et al., 2019), which are developing algorithmic phenotyping technologies. We will describe and analyse the concepts of *digital biomarkers* and *computational phenotyping* that underlie these projects, explain how they are linked to other research in digital phenotyping and compare and contrast some of their epistemological and ethical implications.

## Digital biomarker and computational phenotyping research: two projects

### Digital biomarker research: the RADAR-AD project

The ‘Remote Assessment of Diseases and Relapse – Alzheimer’s Disease’ (RADAR-AD) project is funded by the Innovation in Medicine Initiative, a European Community programme that supports public–private partnerships to address medical challenges. The project aims to explore the potential of digital technologies to improve the assessment of functional decline in early Alzheimer’s disease (AD). It involves about 220 study participants across the AD spectrum in different European countries. Each participant is involved in the study for a period of 70 days and is provided with mobile phones and wearable sensors and is assigned tasks to perform daily. In a second and third stage data is also collected from home-based sensors provided to participants (Tier 2) and in smart homes where participants are invited to stay (Tier 3). Collected data are imported to a platform and analysed to identify patterns of functional decline and test their accuracy against patterns identified through current methods of data collection, such as pen-and-paper questionnaires.

Ultimately, the project aims to identify *digital biomarkers* in people living with AD. The concept of ‘biomarker’ is key to AD research: biomarkers are measurable indicators of a biological state or underlying condition. Patients presenting with molecular biomarkers of AD (for example, the presence of specific proteins, such as amyloid beta, in their brain or certain genetic variants) have an increased risk of developing the condition. Building on this, the concept of ‘digital biomarkers’ refers to the idea that digital devices can help researchers to identify measurable behavioural/cognitive indicators of functional decline. Mobile phones and wearable/home-based sensors enable researchers to monitor aspects of people’s everyday lives that in the mid-term may help in measuring drug effectiveness in clinical trials and, in the long run, can help deliver an AD diagnosis and prognosis. For example, digital devices can be used to collect data on word-finding skills, planning skills, or memory decline through active tasks or games. In addition, sensors embedded in these devices enable a passive collection of data on spatial navigation, sleep quality, interpersonal interaction, and gait. All these factors are considered to be key indicators of functional decline and, therefore, are key to AD prognosis, diagnosis and drug development.

Digital biomarkers are technology-enabled, quantitative and sensitive measures of functional decline in people with early stage AD. Their potential for AD research consists in offering a way to identify functional decline before the onset of known symptoms. This is considered important for a number of reasons. First, tracking functional decline helps to identify at risk populations before the onset of symptoms. This can be seen as a benefit for healthcare systems that can plan care in advance. Second, identifying functional decline at earlier stages of disease and being able to track disease progression helps to develop treatments for early stage disease before symptoms appear. Finally, each individual will likely have different trajectories of functional decline. Validating measures to track functional decline for individuals enables an assessment of the benefits of a treatment at the individual level.

Building on the concept of digital biomarkers and their relevance for AD research, the RADAR-AD project aims to use remote monitoring technologies to accurately and continuously track and monitor clinical changes to address the lack of a robust way to identify symptoms and track disease progression. The immediate goal is to provide a reliable measure of functional decline that can be used in clinical trials for AD drug development. Furthermore, remote monitoring technologies are considered easier to use and less expensive compared with currently available prognostic biomarkers for Alzheimer’s disease, such as lumbar puncture or PET scans, which are very invasive and costly and therefore inappropriate for use in screening programmes to identify at-risk groups

### Computational Phenotyping research: The Minerva Initiative

Computational phenotyping (CP) promises to improve the categorisation of rare disorders. This technology uses facial recognition (FR) algorithms to identify (and classify) the phenotypic features – usually facial dysmorphology – associated with different (genetic) disorders. Machine learning algorithms are trained using digitised facial images of people with a clinical or molecular diagnosis of a rare disease (e.g. Williams syndrome) (Ferry et al., 2014). The *Minerva Initiative* (*MI*) is a research data resource (Minerva Image Resource) and an international consortium of commercial, clinical and academic researchers (Minerva Consortium) involved in CP research (Nellaker et al., 2019). MI’s aim is to facilitate this research by providing opportunities for research collaboration and constructing a secure platform for the sharing of digitized facial image data for use in the training of phenotyping algorithms.

Computational phenotyping algorithms are expected to facilitate rare disease research in a number of ways (Zemojtel et al., 2014). First, CP helps researchers to identify new syndromes by providing a more precise and comprehensive characterisation of symptoms. Second, it supports automated patient ‘matchmaking’ for ultra-rare and currently unknown disorders, enabling researchers to more easily compare and contrast patients’ dysmorphology. Finally, by identifying phenotypic correlates, CP assists researchers in the classification of (causative) variants identified during genomic sequencing.

While CP is an important research tool, this technology is also used in the clinical diagnosis of rare genetic diseases. The subspecialty of dysmorphology is concerned with rare diseases that manifest in early life, impair physical development and may be associated with neurological and cognitive deficits. Diagnosis in dysmorphology involves identifying physical features, including facial features, as markers of an underlying syndrome, which may be genetic in origin (e.g. Angelman’s syndrome) or caused by a prenatal environmental insult (e.g. Foetal Alcohol Spectrum Disorder). Until recently, diagnosis was based upon clinical judgement and relied upon physical examination, photographic evidence and other clinical data, this is now supplemented by molecular diagnostics – karyotyping/exome/whole genome sequencing. Bearing in mind that rare diseases occur infrequently in populations, clinical phenotyping of rare dysmorphological syndromes is regarded as an expert skill, which relatively few clinicians develop (Reardon and Donnai, 2007). Consequently, the diagnosis of dysmorphological syndromes has traditionally involved small groups of experts – (e.g. clinical geneticists specializing in dysmorphology and specialist paediatricians) – meeting in multidisciplinary case conferences to review a patient’s case and agree upon a diagnosis.

The introduction of genomic sequencing into the diagnostic pathway in recent years has meant that case conferences are not always required to diagnose rare syndromes. The evolution of dysmorphology diagnosis from a dependency upon clinical phenotyping based on expert judgement to genotype-assisted diagnosis has been swift, and it is speculated that the introduction of CP into the diagnostic pathway will push this to another level. CP will not only circumvent the need for specialist case conferences in some cases, but also molecular diagnostics, and thus, it is claimed CP will expedite diagnosis at the same time as improving diagnostic yield in dysmorphology (Nellaker et al., 2019). In summary, from a clinical perspective CP technologies offer the potential for accelerated diagnoses in paediatrics, enabling more patients and their families to access clinical and/or social care more quickly.

## Discussion

Both of the above projects assume that digital/computational tools enable the study of phenotypic aspects of disease and will open new opportunities for healthcare and health research. An underlying assumption seems to characterise both approaches: the need to move from an *omics paradigm* to a *phenotyping paradigm* and use digital technologies to ensure more objective data collection and analysis.

Since the latter half of the twentieth century, genomics and other *omic* sciences have been at the forefront of health research and, more latterly, clinical practice, promising to unveil the secrets of disease processes by using technologies that focus on molecular processes. The *phenotyping paradigm,* in contrast, works from the top down to focus on factors, such as facial features, social and cognitive behaviours or lifestyle habits, as a key to identify disease patterns and underlying biological mechanisms.^1^ To date, the identification of these (observable) complex traits has been based upon patients’ recollections and/or healthcare professionals’ assessments. In other words, phenotyping has been seen as dependent upon subjective judgment and therefore, is often assumed to be unreliable or biased.

Digital and computational technologies offer tools to address this issue by enabling (seemingly) more reliable and objective data collection and pattern recognition. As it has been frequently pointed out, however, algorithms are neither value-neutral nor free from human assumptions and judgements. Bias derived from the use of unrepresentative training sets is commonly acknowledged as an issue in algorithmic decision-making, as is the fact that using digital devices for data collection may discriminate against those who have less digital awareness and thus, further reinforce sampling biases. However, despite these obvious shortcomings these technologies are frequently portrayed as supporting more objective phenotyping which will, it is argued, advance current research and clinical practice.

In spite of sharing some underlying assumptions concerning the role of digital technologies in enabling more objective measurement of observable traits, the role played by phenotyping in RADAR-AD and the MI is different. In the mental health domain, the notion of *digital phenotyping* is driven by the technical possibility of seamlessly harvesting digital data produced through online activity and the use of mobile phones and wearable/environmental sensors on a continuous basis. Data are expected to provide precise, continuous and spatially situated information that can advance biomedical research by shifting our understanding of disease in a way that takes into account the lived experience of patients in their natural environment. In a similar vein, the notion of a *digital biomarker* suggests that digital technologies collect objective data over time to track presymptomatic functional decline in more accurate ways because they do not need to rely on patients’ recollection. The collection of digital biomarkers involves data collection through wearable sensors continuously tracking people’s everyday life activities, *computational phenotyping,* in contrast, involves the processing of facial image data – digitised photographs – that are collected on a one off basis (or occasionally as the patient develops) with active consent in a clinical context. In this sense, ‘digital’ versus ‘computational’ phenotyping refer to different ‘affordances’ of information and communication technologies.

The concept of ‘affordance’ was initially outlined in Gibson’s cognitive theory of perception (Gibson, 1986), which explains how human beings engage with their environment. A typical example for Gibson is that a cave is perceived by humans as affording shelter rather than as a cavity in the stone. The concept of affordance has been adopted within science and technology studies and design studies, where it is used to theorise the relationship between user perceptions, the materiality and functionality of the technology and the intentions of technology designers (Nagy and Neff, 2015). As such, *affordance* brings together the material and social dimension of socio-technical systems, and can help us explore how conceptual differences between these two phenotyping projects link material and functional aspects of digital technologies with social practices and potentialities of use.

In the case of RADAR-AD, the project focuses (and capitalises) on the fact that some digital technologies afford portability; they can be carried around, and so can continuously (and passively) collect data about the mundane aspects of people’s lives, which were previously unavailable to researchers and clinicians. Alternatively, in the MI project the focus is on the computational power of algorithmic processing, which means it affords the comparison of large amounts of data gathered from across the world thus, enabling the identification of (unknown) syndromes and/or the causative nature of associated genomic variants.

The reliance on the different affordances embedded within these different technologies convey different expectations of phenotyping in these two projects. When we start exploring the expected affordances of these technologies, it emerges that the assumption of increased objectivity that we have highlighted in both projects has different connotations and materialises in different social practices in RADAR-AD and MI. In the case of RADAR-AD, while the use of wearable digital technology means that the data collected for AD diagnosis may no longer be affected by patients’ subjective recall and associated biases, data collection activities are still dependent upon patient acquiescence and participation; the study participants need to wear different types of sensors, operate them, sometimes engage in games and activities, and may have to engage their families and friends in these assessments. Thus, it is important to emphasise that although some phenotypic data are passively collected from RADAR-AD study participants/patients are also involved in laborious practices to facilitate data collection. This implies that the original expectation of providing more objective data collection needs to be approached with caution, because patients (and their subjectivities) are still very much involved in data collection activities.

Computational phenotyping, by relying on the data analysis affordance of digital technologies, bypasses the challenges of subjective judgement in a different way. Up until now the diagnosis of rare genetic diseases has involved clinicians’ interpretation of phenotypic and/or genotypic biomarkers as evidence of particular disorders. It is argued by some that the introduction of computational phenotyping tools can overcome some of the subjectivity of clinical diagnosis, and its reliance on experience and tacit knowledge. It is anticipated that the use of computational phenotyping tools will enable the mainstreaming of expert diagnostic skills, removing them from the exclusive control of a few clinicians, allowing us to overcome the knowledge deficits of mainstream practitioners and the subjective biases of a small group of healthcare professionals. This is considered as a potential clinical benefit as it may shorten the diagnostic odyssey for patients and their families. At the same time, while a small group of clinicians’ diagnostic judgements form the basis of the CP algorithm training, these are not necessarily available to those who will use the technology to provide a diagnosis and therefore, in some sense, the use of CP tools means that ‘clinical’ expertise is less available to patients and families. Arguably, as in the case of RADAR-AD, the idea that technology can, or will, overcome the problem of subjective judgement needs to be reconsidered within the context of the specific medical and epistemic practice of use.

What can we learn from the comparison of these projects? First, we would argue that looking at the different types of knowledge produced by digital and computational phenotyping tools is important, as it facilitates our understanding of differing societal expectations of these technologies. Second, we believe that disentangling the link between a technology’s functionality and the practices it is expected to impact (or improve) may enable us to explore its social desirability. For example, the fact that wearable sensors still require participants/patients to play an active role in data collection to establish the existence of digital biomarkers, raises questions about the consequences of this (often invisible) labour on research and diagnostic practices: such as, will all users have the skills and confidence to operate these technologies? If they do not, what impact will this have on data representativeness and thus, on the reliability of diagnosis generated by the tool? Alternatively, the use of computational phenotyping tools in the diagnosis of rare disease may change the ways in which the practise of diagnosis is conceptualised: from being a human skill based on tacit knowledge and intuition to an objective algorithm-generated decision. While this may increase the availability of diagnosis, it also carries some healthcare costs. Many argue that computational phenotyping tools should only be used to augment human decision-making within the dysmorphology clinic, indeed, that is how they are currently marketed, however, we predict that there may come a time when it will no longer be regarded as essential to retain the human-in-the-loop in every case, and this will change the diagnosis and practice of dysmorphology and the skillset of this group of experts.

In conclusion, digital phenotyping is a growing phenomenon in research and healthcare, but, as we have demonstrated, its impact and meaning varies. Although this technology may be used in different ways, different uses raise some common issues, which should be acknowledged going forward. Specifically, we draw attention to the ways in which datasets that are used to train algorithms are sourced. Repurposed and new datasets are not only influenced by data collectors’ subjectivities, but also are shaped by data collection infrastructures and technologies. The latter not only influence how data is collected, but what data are collected and used in training and thus, afford particular algorithmic inferences. If we are not careful, it will be non-medical factors, such as, the form and structure of the datasets used in training phenotyping algorithms that will shape the nature of future medicine by defining disease, or what is to be deemed as ‘normal’ or ‘pathological’, and this raises ethical issues such as accountability, transparency and trustworthiness.

## Notes

1. In reality as far as diagnosis is concerned this division is not so clear cut, in the sense that often diagnosis involves the gathering of a range of evidence/data – (gen)omic and phenotypic information. Thus, the diagnostic process in dysmorphology, for example, often involves a movement between genotyping and phenotyping in which one type of data confirms or contradicts a differential diagnosis.

## References

[bibr1-20539517211062885] Ferry Q Steinberg J Webber C , et al. (2014) Diagnostically relevant facial gestalt information from ordinary photos. Elife. 24;3:e02020 DOI: 10.7554/eLife.02020. PMC4067075 24963138

[bibr2-20539517211062885] Gibson JJ (1986) The Ecological Approach to Visual Perception. New York, NY: Taylor & Francis.

[bibr3-20539517211062885] Huckvale K Venkatesh S Christensen H (2019) Toward clinical digital phenotyping: A timely opportunity to consider purpose, quality, and safety. npj Digital Medicine 2: 88. 31508498 10.1038/s41746-019-0166-1 PMC6731256

[bibr4-20539517211062885] Muurling M de Boer C Kozak R , et al. (2021) Remote monitoring technologies in Alzheimer’s disease: Design of the RADAR-AD study. Alzheimer’s Research & Therapy 13(1): 89. 10.1186/s13195-021-00825-4 PMC8063580 33892789

[bibr5-20539517211062885] Nagy P Neff G (2015) Imagined affordance: Reconstructing a keyword for communication theory. Social Media and Society 1: 2.

[bibr6-20539517211062885] Nellåker C Alkuraya FS The Minerva Consortium XX (2019) Enabling global clinical collaborations on identifiable patient data: The minerva initiative. Frontiers in Genetics 10: 611. 31417602 10.3389/fgene.2019.00611 PMC6681681

[bibr7-20539517211062885] Onnela JP Rauch SL (2016) Harnessing smartphone-based digital phenotyping to enhance behavioral and mental health. Neuropsychopharmacology 41(7): 1691–1696. 26818126 10.1038/npp.2016.7 PMC4869063

[bibr8-20539517211062885] Reardon W Donnai D (2007) Dysmorphology demystified. ADC Fetal and Neonatal Edition 92(3): F225–F229. 10.1136/adc.2006.110619 PMC2675338 17449858

[bibr9-20539517211062885] Waring OM Majumder MS (2020) Introduction to digital phenotyping for global health. In: Celi L Majumder M et al. (eds) Leveraging Data Science for Global Health. Cham, Switzerland: Springer International Publishing, pp.251–261.

[bibr10-20539517211062885] Zemojtel T Köhler S Mackenroth L , et al. (2014) Effective diagnosis of genetic disease by computational phenotype analysis of the disease-associated genome. Science Translational Medicine 66(252):252ra123. doi: 10.1126/scitranslmed.3009262. PMC4512639 25186178

